# Expression of a fungal ferulic acid esterase in suspension cultures of tall fescue (*Festuca arundinacea*) decreases cell wall feruloylation and increases rates of cell wall digestion

**DOI:** 10.1007/s11240-017-1168-9

**Published:** 2017-02-07

**Authors:** Phillip Morris, Sue Dalton, Tim Langdon, Barbara Hauck, Marcia M. O. de Buanafina

**Affiliations:** 1 Institute of Grassland and Environmental Research, Plas Gogerddan, Aberystwyth, Wales, UK; 2 Institute of Biological, Environmental and Rural Sciences, Aberystwyth University, Plas Gogerddan, Aberystwyth, SY23 3EB Wales, UK; 3 Department of Biology, 208 Mueller Laboratory, Pennsylvania State University, University Park, PA 16802 USA

**Keywords:** Grass suspension cultures, Cell wall, Phenolic composition, Ferulic acid esterase, Biodegradability

## Abstract

In the cell walls of grasses ferulic acid is esterified to arabinosyl residues in arabinoxylans that can then undergo oxidative coupling reactions to form ferulate dehydrodimers, trimers and oligomers which function to cross-link cell-wall polysaccharides, limiting cell wall degradability. Fungal ferulic acid esterase can release both esterified monomeric and dimeric ferulic acids from these cell wall arabinoxylans making the cell wall more susceptible to further enzymatic attack and increasing cell wall degradability. Non-embryogenic cell suspension cultures of *Festuca arundinacea* expressing a *Aspergillus niger* ferulic acid esterase (*faeA*) targeted to either the apoplast, or endoplasmic reticulum under the control of a constitutive actin promoter, or to the vacuole under the control of a soybean heat shock promoter, were established and FAE activity determined in the cells and medium during a growth cycle. Analysis of the ester-linked ferulates of the cell walls showed that all three transformed cell lines had both reduced ferulate levels and increased levels of xylanase mediated release of wall phenolics on autodigestion as well as increased rates of cell wall digestion in a simulated rumen environment, when compared to control non-transformed cells.

**Electronic supplementary material:**

The online version of this article (doi:10.1007/s11240-017-1168-9) contains supplementary material, which is available to authorized users.

## Introduction

Interest in the relationship between bound cell wall phenolics and their effects on the digestibility of plant material has interested plant scientists for many years. Van Soest (1967) first showed that the digestibility of the cell walls from a number of forages changed with changing phenolic content and Hartley (1972) first showed a significant correlation between the digestibility of cell walls from perennial ryegrass and the p-coumaric/ferulic acid ratio. Hydroxycinnamic acids (HCAs) (*p-*coumaric, ferulic and sinapic acid) have therefore long been known to be bound within the cell walls of many plant species. However, of these, ferulic acid has been found to be the most prevalent in most grass cell walls (Fry and Miller 1989; Grabber et al. 1998; Brett et al. 1999). Ferulic acid residues are attached to cell wall arabinoxylans of grasses via the C5-hydroxyl of α-l-arabinose side-chains on the xylan polymers (Grabber et al. 1998), and estimates have suggested the levels of ferulic acid in grass cell walls can be as high as 3% dry weight (Kroon et al. 1999).

These feruloylated arabinoxylans are believed to be synthesised in the Golgi system prior to export to the apoplast (Brett et al. 1999). Peroxidase-mediated oxidative coupling between ferulic acid residues can then result in the formation of diferulate bridges between the cell wall polysaccharides (Ralph et al. 2004), resulting in the alteration of a large number of characteristics of the cell wall, including accessibility, extensibility, plasticity, adherence and digestibility (Fry and Miller 1989). Such cross-links function to increase structural integrity to the cell wall as well as preventing cell wall degradation by microbial action (Iiyama et al. 1994), although in some circumstances, the plant growth regulator gibberellin may function to increase cell elongation/expansion by lowering the apoplastic peroxidase activity (Fry 1979).

The creation of phenolic cross-links may also occur at a low level as a response to high UV radiation, and evidence from Grabber et al. (1995) shows that the spatial orientation of the feruloylated arabinoxylans is optimised to allow maximum opportunity for cross-linking, and that the level of cross-linking is limited by the availability of hydrogen peroxide. This strongly suggests that the formation of the diferulate cross-links is a highly organised and regulated process. It has been suggested by Carpita (1996) that the HCAs in grass cell walls take the role of hydroxyproline rich glycoproteins that function to cross-link the cell walls in dicots.

Grabber et al. (1998) have also shown that diferulate cross-links can have dramatic effects on the digestibility of plant material. There work on non-lignified maize cell cultures showed that chemically increasing ferulate dimerisation from 18 to 40% caused a very significant reduction in the rate of release of xylans, cellulose and pectins by cell wall degrading enzymes. Varying the degree of ferulate substitution however did not affect digestibility per se, but significantly, the initial rate of digestion was reduced far more than the end point extent of digestion.

Ferulic and p-coumaric acids are also believed to be involved in ether linkages to lignin (Lam et al. 1995), where ferulic acid can cross-link cell wall polysaccharides and lignin as a result of being ether bonded to lignin and ester linked to cell wall polysaccharides, and some evidence suggests that the nature of the bonds between cell wall ferulates and lignins can only occur when ferulate is bound to monolignols (Hatfield et al. 1999). Arabinoxylans are therefore both feruloylated and p-coumaroylated and evidence for this has been produced via the enzymatic hydrolysis of the xylan backbone to produce phenol-carbohydrate conjugates including FAX [3-0-(5-0-feruloyl-*α*-l-arabinofuranosyl)-d-xylose], FAXX {4-0-[3-0-(5-0-feruloyl-α-l-arabinofuranosyl)-β-d-xylanopyronosyl]- d-xylose} and the corresponding p-coumaroyl esters (PAX and PAXX) (Wallace and Fry 1994).

Removing labile phenolics by chemical treatment with alkali is known to increase the biodegradability and nutritional value of low-quality feed such as cereal straw, (Kroon et al. 1999; Bartolomée et al. 1995; Grabber et al. 1998; Faulds and Williamson 1993), and is employed commercially for feed upgrading. It has also been shown that ferulic acid esterase A from cellulytic fungi such as *Aspergillus niger* releases both ferulic acid monomers and dimers from grass cell walls and that this is greatly enhanced by endo-xylanase (Bartolomée et al. 1995, 1997; Faulds and Williamson 1995; Grabber et al. 1998).

Chemically reducing phenolic cross-linking of cell wall carbohydrates is therefore a predictable way of improving the rate of digestion and digestibility of ryegrass. An alternative, however, would be to use genetic modification to reduce the levels of HCAs in the cell walls available for crosslinking either by directly disrupting ester bonds linking phenolics and lignins to cell wall polysaccharides or by preventing excessive feruloylation of cell wall carbohydrates prior to their incorporation into the cell wall.

Progress in tissue culture and genetic transformation of grasses in the last decade has recently been reviewed (Giri and Praveena 2015). However, transformation of *Festuca arundinacea* via microprojectile bombardment of embryogenic cell suspension cultures dates back to the late 1990s (Spangenberg et al. 1995; Kuai et al. 1999), but despite these early reports their are only a few examples of genetic modification of forage quality or plant performance in tall fescue.

Forage quality has been improved by reducing the lignin content and lignin composition of tall fescue plants by down regulating expression of cinnamyl alcohol dehydrogenase and caffeic acid* O*-methyltransferase, resulting in increased in-vitro dry matter digestibility without significant changes in cellulose, hemicellulose or neutral sugar composition, or in p-coumaric acid or ferulic acid levels (Chen et al. 2003, 2004).

Abiotic stress such as oxidative damage or sodium toxicity has been reduced in tall fescue either by over expression of an *Arabidopsis* 2-Cys peroxiredoxin with both peroxidase and chaperon function, where it protected leaves from oxidative damage, probably due to chaperon activity (Kim et al. 2010) or by over expression of *Arabidopsis* Salt Overly Sensitive (SOS) genes, which increased activities of superoxide dismutase, peroxidase and catalase, and the proline content of plants resulting in enhanced salt tolerance and superior plant growth (Ma et al. 2014). Biotic stress in tall fescue plants has also been improved by expression of a shrimp antimicrobial peptide (penaeidin 4-1), which conferred resistance to brown patch disease (Zhou et al. 2016).

Various types of FAE from different fungal species have been expressed in-planta in recent years with varying degrees of success. An A type *Aspergillus nidulans* FAE, when targeted to the apoplast in *Arabidopsis thaliana* reduced cell wall feruloylation and increased enzymatic saccharification of acid-pretreated biomass and plants showed no visible phenotype, but had decreased amounts of wall-associated extensins, and increased susceptibility to *Botrytis cinerea* (Pogorelko et al. 2011; Reem et al. 2016). A type B FAE from *A. niger* targeted to the apoplast, endoplasmic reticulum or vacuole in alfalfa was shown to modify cell wall composition with a reduction in ester linkages with no visible plant phenotype, but with elevated lignin, resulting in recalcitrance to digestion by mixed ruminal microorganisms (Badhan et al. 2014). However, grass cell wall architecture is much more dependent upon the incorporation of ferulates than in *Arabidopsis* or alfalfa, which contains relatively low levels of ferulate.

In grasses, transgenic wheat accumulating heterologous *A niger* type A FAE in the endosperm showed an increase in water-extractable arabinoxylan and a decrease in monomeric ferulic acid, but had shrivelled low weight grain (Harholt et al. 2010). Transgenic *Brachypodium* expressing *A. nidulans* FAE also showed reductions in monomeric and dimeric ferulic acids but increased susceptibility to *Bipolaris sorokiniana* and increased expression of several defense-related genes (Reem et al. 2016).

We have previously reported the effects of expressing *faeA* from *A. niger* in transgenic plants of *Festuca arundinacea* with FAE constitutively or inducibly targeted to the vacuole. Higher levels of expression were however found with inducible heat-shock and senescence promoters (Buanafina et al. 2008). Following cell death and subsequent incubation, vacuole-targeted FAE resulted in the release of both monomeric and dimeric ferulic acids from the cell walls, and this was enhanced several fold by the addition of exogenous β-1,4-endoxylanase. Most of the FAEA-expressing plants showed increased digestibility and reduced levels of cell wall esterified phenolics relative to non-transformed plants (Buanafina et al. 2008). FAE was also targeted to the apoplast, ER and Golgi in order to disrupt feruloylation of the growing cell wall (Buanafina et al. 2010). Plants with lower cell wall ferulate levels, which showed increased digestibility and increased rates of cellulase-mediated release of fermentable sugars, were identified and targeting FAE to the Golgi was found to be more effective than targeting to the endoplasmic reticulum (ER). However reducing the overall level of esterified cell wall HCAs was found to increase the vulnerability of tall fescue to insect herbivory by the fall armyworm (*Spodoptera frugiperda*) (Buanafina et al. 2012).

In this work we report the effect of expression of an *A. niger* FAE gene targeted to the vacuole, apoplast or ER on the levels of cell wall ferulates of cell suspension cultures of the forage grass *Festuca arundinacea*, and subsequent effects on cell wall digestion in the absence of the complication of lignin affecting cell wall digestibility, as well as attempts to further increase the levels of FAEA expression. The work therefore describes studies at the cell culture level aimed at establishing functionality and testing different strategies for achieving targeted expression.

## Materials and methods

### Vector construction

A genomic clone of ferulic acid esterase (*faeA*) from *A. niger* (de Vries et al. 1997) was provided by Dr Ben Bower (Genencor Inc) and used for the construction of vectors in pCOR105 plasmids (McElroy et al. 1990) under the promoter plus 5′-untranslated region of the rice actin gene as described previously (Buanafina et al. 2008). Appropriate sequences were added either to the N-terminus or C-terminus of the *faeA* gene to confer apoplast, vacuole or ER targeting. The N-terminal signal sequences used were the native *Aspergillus* sequence of *faeA* for apoplast targeting, or a mutated (NPIR to NPGR) barley aleurain signal sequence (Rogers et al. 1985) for ER targeting.

For the C-terminals two different sequences were utilized, the native *Aspergillus* end (-GACTW) or a linker (PVAAA) followed by the KDEL reading frame for the ER vector (Table 1). In order to determine whether the level of FAEA expression was limited by enzyme toxicity when expressed under the control of the constitutive promoters, an inducible promoter replacement of the rice actin promoter was made with the soybean 23-kDa 2019E heat-shock gene promoter from pMA406 (Ainley and Key 1990), to generate plasmid pTT3. This promoter when fused to GUS had previously been shown to be inducible by a 10 °C heat-shock and show stable expression for 24–48 h in cell cultures of *Festuca* (Andy Bettany personal communication).

**Table 1:** Vector components

Vector	Promoter	N Signal sequence	C terminus	Target	HygR
pTR2	Actin	*Aspergillus*	-GACTW	Apo	+ (d)
pTU5	Actin	ALE- NPGR	-PVAAAKDEL	ER	–
pTT3	HS	ALE- NPIR	-GACTW	Vac	+ (d)

ALE-NPGR is a barley aleurain signal sequence with an altered vacuolar targeting motif (NPIR to NPGR). The COOH peptides are the native FAE end (-GACTW), a linker and synthetic ER targeting motif (-PVAAAKDEL). Expected location of the expressed FAE is vacuolar (VAC), endoplasmic reticulum (ER) or apoplast (APO). Presence or absence of hygromycin resistance cassette indicated by − or +, (d) indicates divergent transcription from FAE

For stable transformations vectors were finally used with FAE driven either by the rice actin promoter, (pTU5, pTR2,) or the soybean 2019E 23 kD heat shock promoter (pTT3) with either an intact barley aleurain vacuolar targeting sequence, (pTT3), or with modified versions for apoplast targeting (NPIR to NPGR) and with modification to incorporate the KDEL sequence for ER retention (pTU5) or containing the native *Aspergillus* excretion signal for apoplast targeting (pTR2).

Vector pTU5 was co-transformed with a hygromycin (*hph*) resistant gene driven by a CaMV35S promoter (pROB5) in pUC18 (Bilang et al. 1991), while pTR2 and pTT3 vectors co-integrated a Hind*III* cassette derived from pTRA151 (Zheng et al. 1991), containing the hygromycin resistance gene (*hph*) under the control of a CaMV35S promoter. Further details of vector construction can be found in Dunn-Coleman et al. (2006).

### Tissue culture and transformation

Cell suspensions of tall fescue (*Festuca arundinacea*), cultivar 20BN3 were initiated from shoot-tip-derived embryogenic callus grown on medium 133 MS pH 5.6, with 3% sucrose and 3 mg l^−1^ 2–4-D solidified with 0.8% agar. Eight to ten weeks old embryogenic suspension cultures were bombarded either with a single co-integration plasmid vector containing FAE and hyg resistance genes, or with a co-transformation vector containing FAE and with plasmid pROB5 conferring hygromycin resistance, using a Particle Inflow Gun (Finer et al. 1992) as in Dalton et al. (1999). Transformants were selected with hygromycin (25–50 mg l^−1^) over a 10–12 weeks selection period at 25 °C under continuous white fluorescent light (60 μE m^2^ s^−1^).

Callus cultures were maintained in Petri dishes on agar-solidified 133 medium, and transgenic calli were grown on the same medium containing 150 units of hygromycin. Cultures were grown at 25 °C and were subcultured every six to eight weeks. Stock suspension cultures of transformed and non-transformed lines were initiated by subculturing of 0.5–1.0 g callus into 50 ml of liquid 133 medium and allowed to reach a density of about 100 g per litre before subculturing into 100 ml medium in 250 ml Erlenmeyer flasks at a dilution of 1:5 (v/v). Transformed cells were grown in medium containing 150 units of hygromycin per litre at 25 °C and shaken at 150 rpm on an orbital shaker and were subcultured every 7 days.

### Molecular analysis of transformed cell cultures

PCR analysis was performed to screen cell culture lines containing the FAE gene. A simple DNA preparation from transgenic and control (non-transformed) cultures was used. Callus tissue was placed on a 5 × 2 cm strip of sterile 180 water proof latex paper (Rhynowet) and ground between the paper using 120 µl of buffer S (100mM TrisHCl, pH 5.0, 100mM NaCl, 50 µM EDTA, pH 8.0 and 2% SDS) and then transferred to 0.5 ml Eppendorf tubes. 80 µl phenol/chloroform was added, vortexed, centrifuged and the aqueous top layer transferred to Eppendorfs containing 70 µl of isopropanol. Pellets were then resuspended in 10 µl TE buffer. PCR analysis was carried out using primers specific for the FAE gene. PCR reaction mixtures of 25 µl volume contained: 0.5 µl of final DNA extract, 48 µM dATP, dTTP, dCTP, dGTP, 0.2 µM primers (FAEN5- CTAAA GCTTA CCATGG CGGCC GCCTC CACGC AGGGC ATCTC CGA and FAE3-TCTAA GCTTG CGGCC GCGCA CGGCC AGGTC CATGC GTCGT CATCCC), 1× Amplitaq commercial buffer (as supplied), 3mM MgCl_2_ and 1U Taq DNA polymerase (Perkin-Elmer). PCR reactions were carried out in a Perkin-Elmer 480 under the following conditions: 1 cycle of 94 °C for 1 min and 30 s, 30 cycles of 30 s denaturation at 94 °C, 30 s primer annealing at 65 °C, 1 min extension at 72 °C. PCR amplification products were analysed by electrophoresis on 1.0% agarose/TAE gels in Tris-borate-EDTA buffer (Sambrook et al. 1989) where the expected size was ~1.2 Kb.

### Southern hybridisation

Genomic DNA was isolated from PCR positive cell cultures following the procedure of Dellaporta et al. (1983) and digested overnight at 37 °C with *Hind*III (which excises the FAE gene) or with *B*g*l*II (which restricts at one site in the plasmid DNA) restriction enzymes (Boehringer-Mannhein). Ten micrograms of digested DNA were separated by electrophoresis through 1.0% agarose gels in Tris-borate-EDTA buffer (Sambrook et al. 1989) and transferred onto Hybond N^+^ membrane by capillary blotting according to the manufacturer’s instructions (Roche-Mannhein). DNA was fixed to the membrane by UV cross-linking, using a CL-1000 ultraviolet crosslinker (UVP). A digoxigenin-labelled FAE probe was prepared by PCR of 100 pg plasmid DNA using primers and conditions described above, except that the final nucleotide concentration was: 48 µM dATP, dCTP, dGTP; 36 µM dTTP, 12 µM DIG-dUTP (Boehringer–Mannhein). The product of the reaction was extracted from a 0.8% low-melting point agarose/TAE gel.

Membranes were pre-hybridised in 6.5 ml EasyHyb solution (Boehinger–Mannehein) at 42 °C for 3–5 h, in a hybridisation oven (Stuart Scientific). The DIG labelled FAE probe, corresponding to the entire 1.2Kb was boiled for 10 min and 10 µl was added to 6.5 ml of fresh, pre-warmed EasyHyb solution and hybridisation was carried out overnight. After hybridisation, membranes were washed in trays: twice in 2× SSC/0.1% SDS for 10 min each at room temperature, then twice in 0.2× SSC/0.1% SDS for 30 min each at 68 °C. Blocking and detection of the hybridised probe DNA was carried out as described by the manufacturer (Boehringer–Mannheim) except for blocking which was for 90 min. Exposure times were 1–2 h.

### Determination of cell growth

Cell growth was determined from increases in fresh and dry weight by sampling 10 ml of suspension from triplicate flasks at each time point and filtering onto pre-wetted Whatman No. 1 filter paper under vacuum. Dry matter determinations were obtained after drying cells at 50 °C to constant weight.

### Determination of ferulic acid esterase activity

Ferulic acid esterase activity was determined in soluble extracts from 0.5 g of fresh or frozen cell cultures. Cells were ground in a precooled mortar and pestle with 0.1 M sodium acetate, pH 5.0 extraction buffer containing 0.5% Triton. Extracts were incubated with 24mM ethyl ferulate (ethyl 4-hydroxy-3-methoxycinnamate) at 28 °C for 24 h. Following incubation, and centrifugation, soluble extracts were loaded onto an activated reverse phase C18 µNova Sep-Pak column (Waters), and eluted with 4 ml 100% methanol (MeOH). FAE activity was calculated from the amount of ferulic acid released determined by HPLC. One unit of FAE activity equals 1 µg ferulic acid released from ethyl ferulate at 28 °C in 24 h.

Heat-shocked cells were assayed in the same way as above, but prior to assay the cells were routinely heat shocked at 38 ± 1 °C for 2 h in a shaken incubator and then allowed to recover for 24 h at 25 ± 1 °C on an orbital shaker. At the end of the 24-hour period cells were assayed for FAE activity by HPLC. FAE activity was also determined by measuring the release of monomeric and dimeric ferulic acids from self-digested cell culture samples.

### Determination of cell wall phenolics

Following exhaustive extraction of soluble phenolics with aqueous methanol, ester bound phenolics were extracted from the cell walls of freeze dried powdered cell cultures (50 mg), with 1 M NaOH (5 ml) followed by incubation at 25 °C for 24 h, in the dark under N_2_. After centrifugation, acidification and precipitation of solubilised carbohydrates with MeOH at 4 °C, the extracted phenolics in the aqueous phase were loaded onto an activated reverse phase C_18_ µNova Sep-Pak column and eluted with 100% MeOH, and analysed by HPLC.

HPLC was carried out on a µNova Pak C18 8 × 10 Radial Compression Module (Waters) with MeOH: 5% acetic acid either with a 35–65% linear MeOH gradient in 15 min (for FAE assay) or with a 30–70% linear MeOH gradient in 25 min (for monomer and dimer cell wall components) at 2 ml/min. Hydroxycinnamic acids were monitored and quantified at 340 nm, with a Waters 996 photo-diode array detector with UV /visible spectra collected at 240–400 nm, and analysed with Millennium software (Waters Inc) against authentic monomer standards, or using response factors for the various dehydrodiferulate dimers reported by Waldron et al. (1996).

### Determination of cell digestibility

An autodigestion procedure was used to investigate the effect of the expression of FAEA on the release of cell wall HCAs on cell death. Callus cultures (0.2–0.5 g fresh wt) were ground in 0.1 M Na acetate extraction buffer pH 5.0, in the presence and absence of excess β-1-4-endoxylanase (1000 U/sample) (GC140 Genencor Inc), without additional substrate, and incubated at 28 °C for 24 to 72 h with shaking at 120 rpm. Following centrifugation, soluble extracts were loaded onto a reverse phase C_18_ µNova Sep-Pak column (Waters Inc), eluted with 100% MeOH and analysed by HPLC as described above.

Digestibility parameters were also determined from in vitro gas production measurements. Freeze dried powdered cell suspension cells (1.0 g samples) were fermented in triplicate in 165-ml capacity serum bottles in 90 ml of bicarbonate buffered medium and were inoculated with rumen micro-organisms in 10 ml of clarified rumen fluid, at 30 °C for 48 h according to the pressure transducer technique of Theodorou et al. (1994). This technique quantified the increase in head-space gas pressure (and thus the gas volume) in closed batch cultures inoculated with rumen micro-organisms. The cumulative gas production profiles were used to calculate the initial rate of gas evolution over the first 6 h, the maximum rate of gas evolution, the time to maximum rate and the total gas volume.

## Results and discussion

### Vector construction and testing

A number of expression vectors in pUC plasmids with *faeA* driven by a rice actin promoter, with a number of C and N terminal ends, (to confer vacuolar, apoplast, or ER targeting), and with modifications to the core ORF, (to enhance expression in plants with the codon choice based on published barley preferences) were made and tested in transient expression for FAE activity following micro-projectile bombardment of grass cell cultures. A smaller number of functional constructs were chosen for stable transformations and used to construct vectors containing constitutive and inducible heat-shock promoters.

Reproducible transient FAE activity was detected in soluble cell extracts of *Festuca* cell cultures with FAXX or ethyl-ferulate as substrates. No FAE activity was detected with the constructs lacking an N terminal signal sequence, irrespective of C terminal modifications and consistently lower FAE activity was detected with the native FAE N terminal signal than with the barley aleurain signal (data not shown). Mutation of the NPIR vacuolar localisation motif of the barley aleurain transit peptide to NPGR was found to be as effective as removal of this motif but both resulted in a decrease in transient FAE activity compared with the native NPIR (additional data are given in Online Resource Fig. S1). Removing the putative glycosylation site or truncating the ORF at the 32 amino acid site also severely reduced FEA expression from the aleurain signal. In some cases the activities of FAE were lower with the native *Aspergillus* C terminal end (CTW) than when a linker sequence was added (CTW-PVAAA) suggesting that the native *Aspergillus* C terminal end has unusual and probably deleterious effects in grasses (data not shown).

The co-integration vectors containing the bacterial gene conferring hygromycin resistance were made following a number of experiments to compare the levels of FAE expression obtained from constructs in which the orientation of FAE and the resistance gene were varied. A tandem arrangement of both genes, with FAE followed by the resistance gene with the same reading frame polarity, was found to be at least as effective as other arrangements in transient expression assays (additional data are given in Online Resource Fig. S2) and was therefore used for the final series of constructs.

### Targeting of FAE expression

**Fig. 1:**
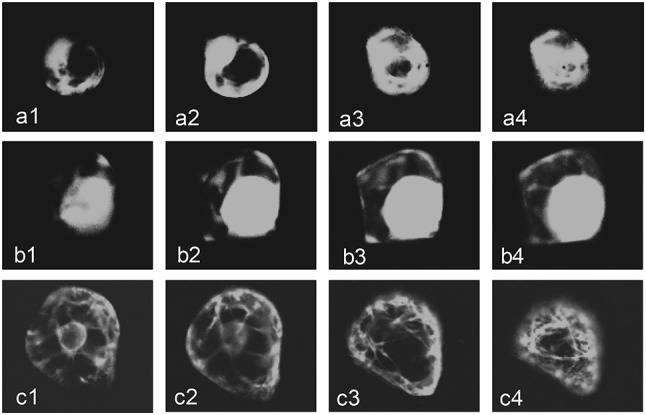
Expression of sGFP targeted to the apoplast (**a1**–**a4**), vacuole (**b1**–**b4**) or ER (**c1**–**c4**) in suspension culture cells of *Festuca arundinacea*. Serial 10-µm confocal fluorescence microscope images were taken through single cells 24 h after bombardment with plasmid DNA. *sgfp* replaced ferulic acid esterase in pTR2 (apoplast), pTT3 (vacuole) and pTU5 (ER)

In order to obtain information on the effectiveness of targeting sequences in delivering FAE to the different compartments, parallel vectors containing a green fluorescent protein (*sgfp*) were constructed and the cellular distribution of gfp fluorescence determined in transient expression studies in embryogenic cell cultures of *Festuca*. Figure 1 shows examples of serial confocal microscopy images through single cells expressing gfp targeted to the apoplast, vacuole or ER, which are not inconsistent with these cellular locations.

### Expression of FAE in grass cell cultures

In order to test the effectiveness of the FAE vectors and obtain early information on gene expression and subsequent effects on cell wall phenolic composition, non-embryogenic cell suspension cultures of *Festuca arundinacea* stably expressing FAE were produced. Cultures were bombarded with different plasmid vectors under the actin promoter with apoplast, vacuolar, or ER, target sequences. A minimum of 20 hygromycin resistant colonies per vector was selected, screened for FEA expression and three FAE expressing lines established as cell suspension cultures.

Line TR2 expressed FAE under the actin promoter targeted to the apoplast using the native *Aspergillus* FAE excretion signal, line TU5 expressed FAE under the actin promoter targeted to the ER using the 5′ NPGR aleurain, and 3′KDEL signal sequences, and line TT3 expressed FAE under the soybean HS promoter targeted to the vacuole using the native 5′ NPIR aleurain signal sequence.

### Molecular analysis

**Fig. 2:**
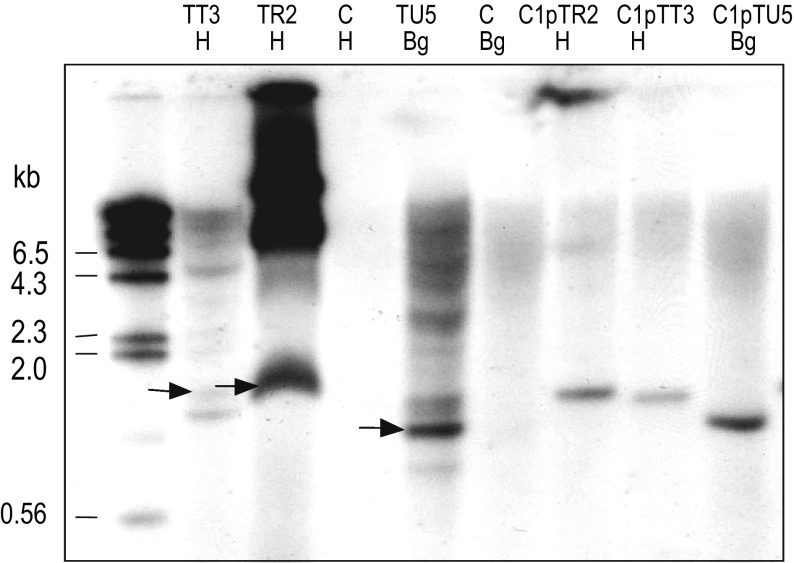
Southern blot hybridisation analysis of transgenic *Festuca arundinacea* callus hybridised with FAE probes. *H = Hin*dIII, *Bg = Bgl*II digests, *C* = DNA from non-transformed control cultures, *C1* = DNA from non-transformed cultures spiked with 1 genome equivalent of the corresponding plasmid.* Each track* contains 14ug DNA. *Arrows* indicate the sizes and locations of expected fragments from pTT3, pTR2 and pTU5 plasmids. Sizes of molecular-weight markers are indicated on the *left*

PCR analysis of transgenic callus from the three lines transformed with pTT3, pTR2 or pTU5 plasmids gave the predicted 1.2 Kb fragment of the FAE gene (data not shown), and further Southern blot analysis hybridised with FAE probes, showed at least one intact copy of FAE in line TT3 and TU5 and multiple intact copies in line TR2 together with a number of higher molecular weight bands probably corresponding to multiple tandem rearrangements of the FAE gene (Fig. 2).

### Growth and cell wall ferulate accumulation in cell suspension cultures

**Fig. 3:**
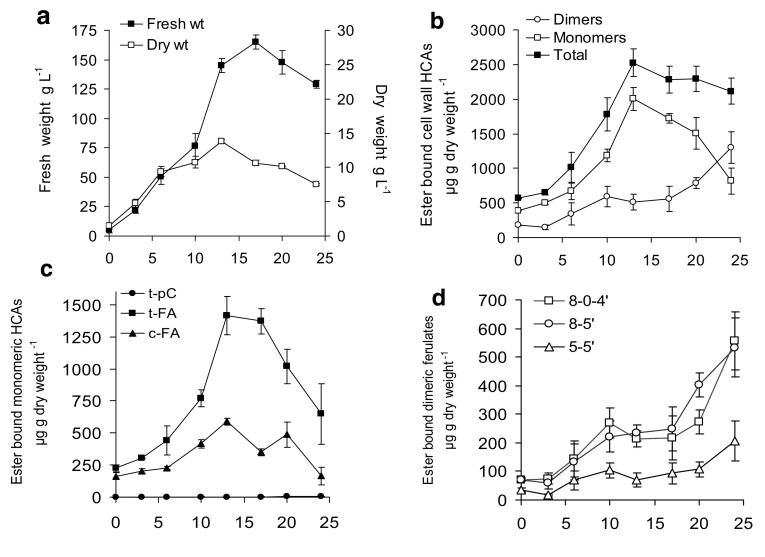
Growth (**a**), monomeric and dimeric ferulate content (**b**) and individual HCA monomers (**c**) or dimer content (**d**) of cell walls during a culture cycle of control non-transformed, non-embryogenic cell suspension cultures. *tFA* = trans-ferulic acid, *cFA* = cis-ferulic acid, 8 -5′ = 8 - 5′diferulic acid, 8-0-4′ = 8-0-4′ diferulic acid, 5–5′ = 5–5′diferulic acid. Mean ± sem (n = 3)

The growth characteristics and HCA content of the cell walls of non-embryogenic cell suspension cultures of *Festuca* was first established throughout a growth cycle. These control cultures showed normal growth kinetics for cell suspension cultures with a typical increase in fresh weight co-incident with a decrease in dry weight at days 10–13 due to cell expansion, which is characteristic of carbon limitation (Fig. 3a). The levels of total ferulate monomers in the cell walls increased in parallel with fresh weight and dry weight increases up to day 10–13, but ferulate dimers increased only slowly and with a 3–5 day lag following subculture. Co-incident with the cell expansion phase, the levels of ferulate monomers rapidly decreased and the levels of ferulate dimers increased, particularly towards the end of the stationary phase of the growth cycle. However there was no significant change in total cell wall ferulates during this period (Fig. 3b). Changes in the levels of the individual monomeric and dimeric ferulates are shown in Fig. 3c, d.

### Heat–shock inducible FAE activity

**Fig. 4:**
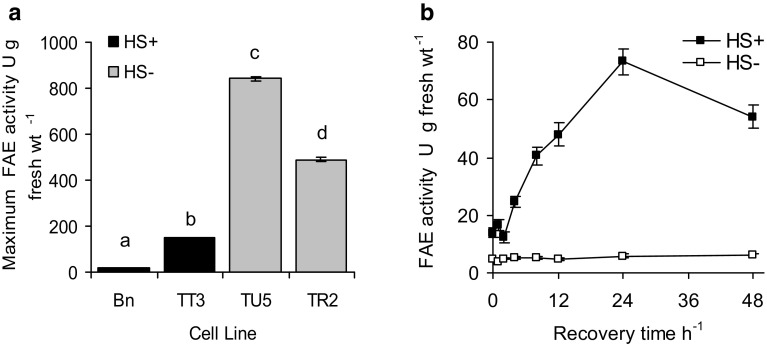
Maximum FAE activities in cell lines (**a**) and kinetics of heat-shock induced FAE activity in 21 day old cell suspension cultures of line TT3 (**b**). Cultures were heat-shocked at 38 ± 1 °C for 2 h and assayed for FAE activity over the following 48 h at 25 °C. Mean ± sem (n = 3). Different letters indicate significant differences between means (p < 0.01)

The kinetics of heat–shock induced FAE activity was determined in 14-day-old cell suspension cultures of *Festuca* line TT3 stably transformed with plasmid pTT3 with FAE targeted to the vacuole. FAE activity was inducible 14-fold with a + 13 ± 1 °C heat-shock for 2 h (Fig. 4a) and was maximal after 24 h recovery at 25 °C (Fig. 4b).

### Growth and FAE expression in transformed cell lines

**Fig. 5:**
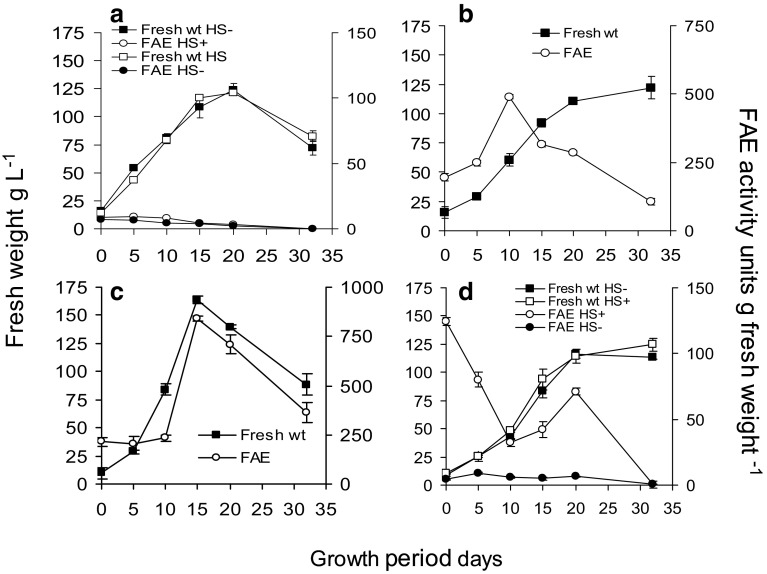
Growth and FAE activity during a culture cycle of cell suspensions of non-transformed control Bn cell line (**a**), and transformed lines TR2 (apoplast targeted FAE), (**b**), TU5 (ER targeted FAE) (**c**) and TT3 (heat-shock vacuolar targeted FAE) (**d**). Cultures were heat-shocked at 38 ± 1 °C for 2 h and assayed for FAE activity after 24 h recovery at 25 ± 1 °C. Mean ± sem (n = 3)

**Fig. 6:**
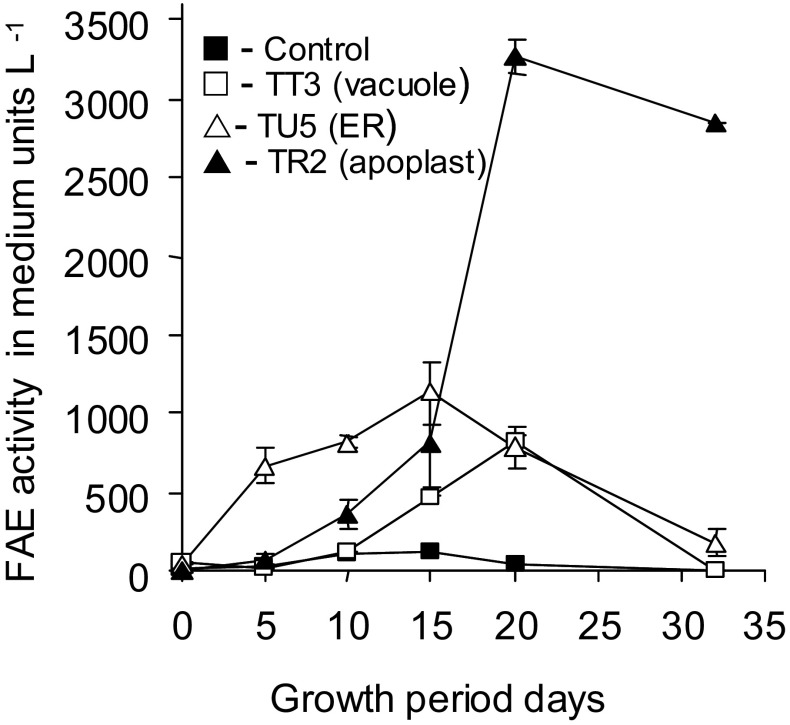
FAE activities in the culture medium during a growth cycle of cell suspension cultures of the non-transformed control Bn cell line and transformed lines TR2 (apoplast targeted FAE), TU5 (ER targeted FAE) and TT3 (heat-shock vacuolar targeted FAE). Mean ± sem (n = 3)

Cell growth, and FAE activity in the cells (Fig. 5) and FAE activity in the media (Fig. 6) were determined during a growth cycle in the three transformed cell lines. The growth kinetics of the transformed cell lines showed similar growth kinetics to that of the non-transformed control line Bn, but had slightly slower growth rates, most likely due to these cells having been previously grown in hygromycin-containing medium.

In the TR2 apoplast targeted cell line with FAE being expressed constitutively, the level of FAE activity increased up to 10 days, mirroring the growth kinetics of the cells, and then, declined well before the cell culture entered stationary phase (Fig. 5b). At this time point FAE activity began to accumulate significantly in the media (Fig. 6), in line with FAE excretion into the apoplast and loss into the surrounding media. However this did not appear to have had any deleterious effects upon the growth of the cells in comparison to the non-transformed cell line.

In the TU5 ER targeted cell line with FAE expressed constitutively the level of FAE activity increased up to 15 days, also mirroring the growth kinetics of the cells, and then declined as the cells entered stationary phase, expanded and lost viability (Fig. 5c). However FAE activity in the medium did not increase indicating that FAE was retained within the cells after cell death (Fig. 6). Further evidence of association of the FAE with intercellular membranes in this line was found in cell residues after extraction of soluble proteins, where little FAE activity was found in the residues of vacuolar or apoplast targeted cells, but significant FAE activity was found in detergent extracts of the residues of cells transformed with ER targeting signals, particularly those which showed the highest activity in soluble protein extracts (data not shown).

In the TT3 cell line with FAE targeted to the vacuole and the gene under the control of an inducible heat-shock promoter, heat-shock inducible FAE activity was maximal in freshly sub-cultured cells and then declined over the following 10 days as cell age increased (Fig. 5d) with little loss of activity into the medium (Fig. 6). A second peak of activity was found from 10 to 20 days with some loss to the medium. FAE activity was not induced by heat-shock in stationary phase cells and heat shock had no effect on cell growth. Furthermore, no FAE activity above background was detected in the absence of heat-shock, at any time throughout the growth cycle of the cells (Fig. 5d). Similarly heat-shock did not induce any increase in endogenous background FAE activity in control Bn cells (Fig. 5a).

### Effects of FAE expression on cell wall phenolic accumulation

**Fig. 7:**
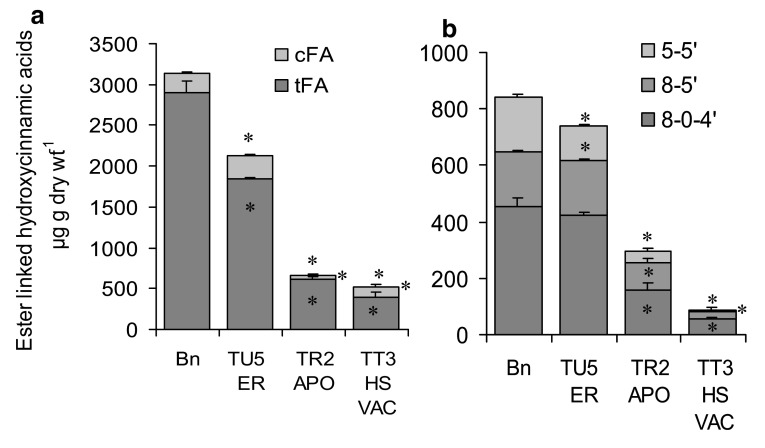
Levels of ester bound monomeric (**a**) and dimeric (**b**) ferulates, extracted from cell walls of stationary phase cell suspension cultures of transformed lines TU5 (ER targeted FAE), TR2 (apoplast targeted FAE) and TT3 (heat-shock vacuolar targeted FAE) compared with non transformed controls cultures (Bn). Ester linked ferulates were released with 1 M NaOH at 25 °C. *tFA* = trans-ferulic acid, *cFA* = cis-ferulic acid, 8 - 5′ = 8 - 5′diferulic acid, 8-0-4′ = 8-0-4′ diferulic acid, 5-5′ = 5-5′diferulic acid. Mean ± sem (n = 3). *Asterisk* indicates significant difference from corresponding control Bn means (student* t* test; p < 0.05)

The major ester linked monomeric and dimeric phenolics of the cell walls of the control non-lignified, non-embryogenic cell suspension culture line Bn were trans- and cis-ferulic acid and the 5–5′, 8 − 5′ and 8-0-4′ ferulate dimers, but unlike the cell walls of *Festuca* leaves, they contained only trace amounts of esterified p-coumaric acid (Fig. 7).

Analysis of the ester-linked phenolics of the cell walls of 21-day-old stationary phase cells showed that all three FAE expressing lines had reduced levels of both monomeric and dimeric ferulates compared to controls. Ferulate monomers were reduced by 80% in the TR2(Apo) and TT3(Vac) lines and by 30% in the TU5(ER) line (Fig. 7a), and ferulate dimers by 90% in the TT3(Vac) line, 60% in the TR2(Apo) and by 10% in TU5(ER) line (Fig. 7b).

The very low levels of ester bound ferulates found in the TT3 line are at first slightly surprising, given the low levels of cellular FAE activity, sequestration of FAE in the vacuole, and the finding of little excretion of FAE into the medium. It should be noted however that the heat-shock induced FAE activities shown in Fig. 5d and in Fig. 6 are transient activities, 24 h post heat-shock, and represent the potential for heat-shock induction of FAE activity and cellular excretion of cells of different ages throughout the growth cycle, whereas the cell wall ferulate levels represent the levels in stationary phase cells which had been repeatedly heat-shocked during the growth cycle (at days 0, 5, 10 15 and 20). Hence the low level of bound ferulates found in the TT3 line may be due to the combined application of the multiple heat-shock treatments.

### Effects of FAE expression on cell wall digestion

#### Release of cell wall ferulates on autodigestion

**Fig. 8:**
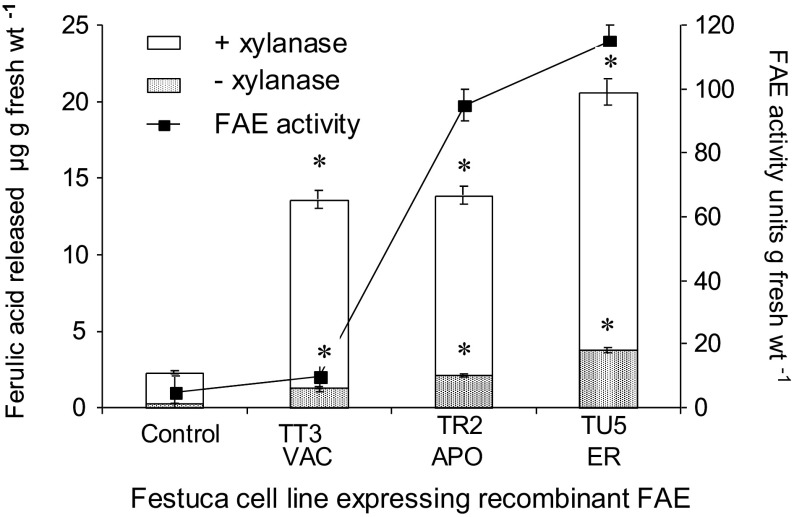
FAE activities and release of ferulic acid from cell walls on cell death by FAE expression and stimulation by β-1-4 endo-xylanase in callus cultures of transformed lines TU5 (ER targeted FAE), TR2 (apoplast targeted FAE) and TT3 (heat-shock vacuolar targeted FAE) compared with non-transformed control Bn cells. *Asterisk* indicates significant difference from corresponding control Bn means (student* t* test; p < 0.05)

Self-digestion of the hemicellulose component of the cell wall after disruption of callus cultures of vacuolar, apoplast or ER targeted FAE-expressing cells resulted in the release of small but significant quantities of esterified ferulic acid at higher levels compared to non-transformed control cultures (Fig. 8). Ferulic acid release was also simulated 7–10-fold in an FAE dependant manner by the addition of exogenous xylanase, which is known to act synergically with FAE in cell wall digestion by rumen microorganisms. Comparing the amounts of ferulic acid released in the presence of exogenous xylanase (Fig. 8), (when converted to a dry weight basis), with the reduced levels of ferulic acid found in the different cells lines (Fig. 7), ferulate release amounted to 0.9% of the total cell wall ferulates for control Bn cells, 12.6% for TU5 (ER) cells, 24.9% for TR2 (Apo) cells and 37.9 for TT3 (HS-Vac) cells.

#### Digestibility parameters derived from in-vitro gas production

Traditionally tissue digestibility is determined as in-vitro-dry matter digestibility (IVDMD) and is an estimate of end point digestion (Jones and Hayward (1973). IVDMD is not however simply a function of the ester linked ferulate or diferulate content of the cell walls but additional factors such as lignin, ferulate oligomers and ether-linked ferulates are also involved in determining endpoint digestibility. More informative estimates of tissue degradability can be obtained from fermentation kinetics by determining rates of gas evolution under rumen-like conditions (Theodorou et al. 1994).

**Fig. 9:**
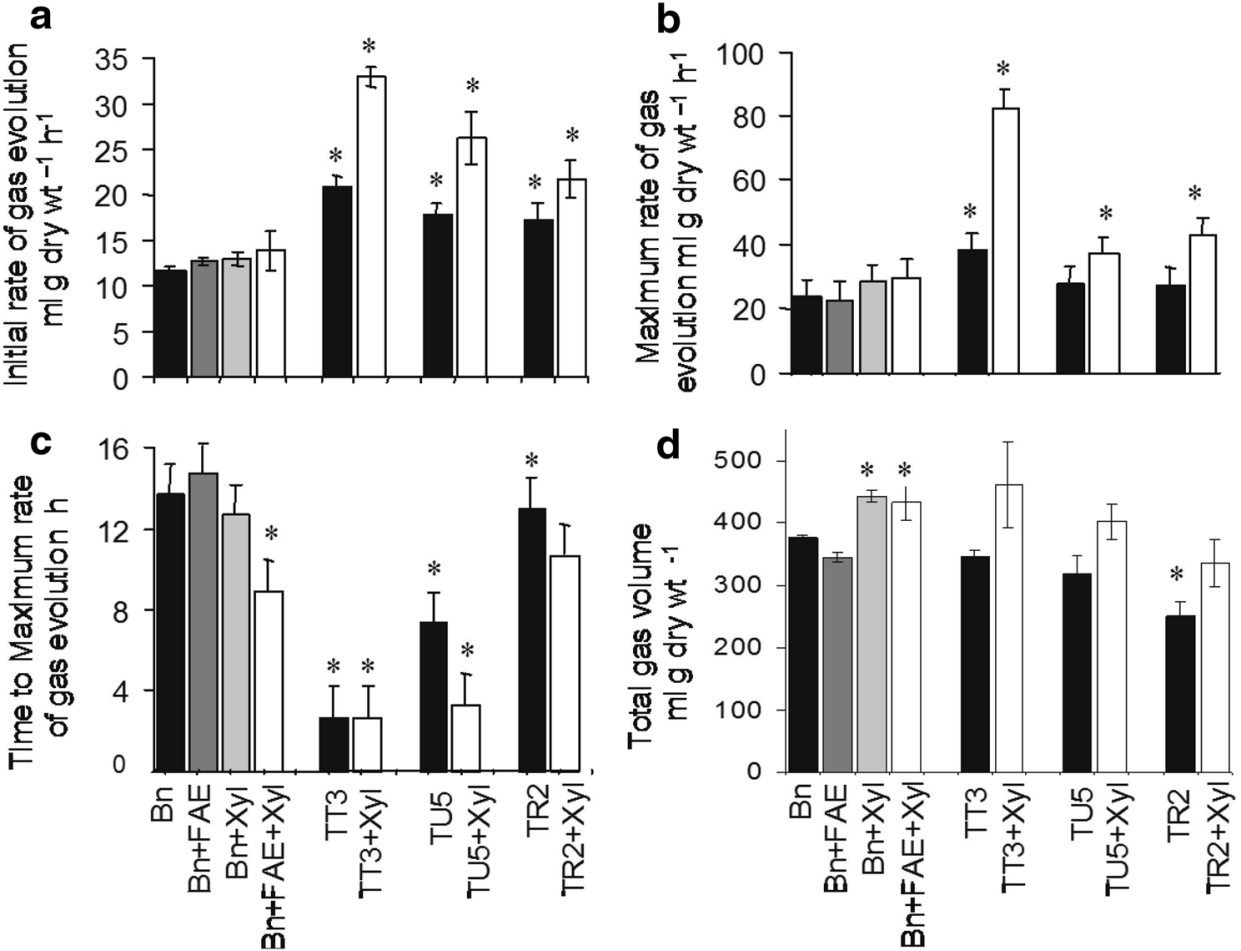
Digestibility parameters of suspension cultured cells derived from gas evolution kinetics and the effects of exogenous addition of FAE and *β* ,1–4 endoxylanase. Initial rate of gas evolution (**a**), Maximum rate of gas evolution (**b**), Time to maximum rate (**c**), End point total gas volume (**d**). Twenty-one day old stationary phase cells of the transformed lines TU5 (ER targeted FAE), TR2 (apoplast targeted FAE) and TT3 (heat-shock vacuolar targeted FAE) were compared with the non-transformed control Bn line. + FAE = + 10 U *Aspergillus niger* ferulic acid esterase, + Xyl = + 1000 U GC140 *Trichoderma reesei β* ,1–4 endoxylanase (Genencor Inc). Mean ± SD of two independent experiments (n = 3 for each experiment). *Asterisk* indicates significant difference from corresponding control Bn means (student t test; p < 0.05)

The initial rate of digestion of control Bn cell walls, as determined from rates of gas evolution was stimulated by 9% by the exogenous addition of 10U *Aspergillus niger* FAE, by 10% with exogenous addition of 1000U GC140 *Trichoderma reesei* β-1-4 endoxylanase and by 19% with exogenous addition of FAE and xylanase combined (Fig. 9a).

Rates of cell wall digestion of the three cell lines expressing FAE were significantly higher than control Bn cells in the absence of exogenous FAE (by 78% in the TT3 cell line, 52% in the TU5 line and 48% in the TR2 line), and were further enhanced by the addition of 1000U β-1-4 endoxylanase (by 58% in the TT3 line, 47% in the TU5 line and 25% in the TR2 line). Furthermore initial rates of digestion in the presence of added xylanase were substantially higher than control cells in the presence of added FAE and xylanase (by 136% in TT3, 88% in TU5 and 56% in TR2) (Fig. 9a), indicating that endogenously produced FAE can effectively substitute for exogenously supplied FAE; although the higher rates of digestion may be primarily due to the lower phenolic content of the walls of FAE expressing cells.

Similar but less pronounced results were found for maximum rates of gas evolution (Fig. 9b). The time to reach maximum rates of degradation was also significantly reduced in cells expressing FAE in the vacuole and ER, which could be only partially achieved with exogenous application of FAE and xylanase to control cells walls (Fig. 9c). However the final total gas volume, which represents the end point in digestion was either unaffected or showed a decrease in digestibility (Fig. 9d), as found previously in end point digestibility studies with highly feruloylated cell walls (Grabber et al. 1998).

The lack of effects on end point digestion, but significant increases in the initial rates of fermentation and reductions in the time to reach maximal rates of fermentation under rumen-like conditions were also found with *Festuca arundinacea* plants expressing FAE (Buanafna et al. 2006, 2008).

### Attempts to increase the level of FAE expression

As indicated earlier, we initially did not attempt to maximise FAE expression levels because of concerns that high FAE activity could disrupt normal cell wall development and result in cell death. As it appeared that low level FAE expression was not lethal and resulted in normal cell growth, additional work was undertaken to increase the levels FAE activity. This centred on two approaches. Firstly establishing if low FAE activity was due to effective transcription but abnormal translation or post-translational modification resulting in ineffective FAE protein or secondly, if low level activity was due to low levels of gene transcription from the promoter. As the actin promoter used to make the initial vectors was subsequently found to contain a 5 bp deletion relative to the published sequence near the NCO splice site, which may affect splicing at the adjacent 3′ site, the original rice actin sequence in this region was restored. A rice repetitive element is also present in the upstream region of the actin promoter and this may have unpredictable effects on vector expression, and was therefore removed. Both these modifications resulted in significantly increased gfp expression in transient assays in *Festuca* cells (additional data are given in Online Resource Fig. S3) and both modifications were incorporated into subsequent FAE vectors for plant transformation (Buanafna et al. 2006, 2008).

In order to study translational effects of FAE expression, sgfp was fused to the 5′ or 3′ end of FAE, and sgfp expression monitored in transient assays. Translationally fused forms of sgfp at the N and C terminus resulted in slightly lower levels of gfp fluorescence whereas a non translated FAE reading frame in the 3′ UTR of sgfp did not affect sgfp fluorescence compared with sgfp controls (additional data are given in Online Resource Fig. S4). This may indicate that poor translation of *fae* may be the cause of low levels of FAE expression, and implies that codon usage of the *A. niger* FAE gene may not be optimal for obtaining high level FAE activity in grasses.

## Electronic Supplementary Material

Below is the link to the electronic supplementary material.

Supplementary material 1 (DOC 5712 KB)

## Abbreviations

FAE: Ferulic acid esterase
HCAs: Hydroxycinnamic acids
FAX: 3-0-(5-0-feruloyl-α-l-arabinofuranosyl)-d-xylose
FAXX: 4-0-[3-0-(5-0-feruloyl-α-l-arabinofuranosyl)-*β*-d-xylanopyronosyl]-d-xylose
PAX: 3-0-(5-0-coumaroyl -α-l-arabinofuranosyl)-d-xylose
PAXX: 4-0-[3-0-(5-0- coumaroyl -α-l-arabinofuranosyl)-β-d xylanopyronosyl]- d-xylose
ER: Endoplasmic reticulum
MeOH: Methanol
ORF: Open reading frame
