# Supplementary material for: Expression of a fungal ferulic acid esterase in suspension cultures of tall fescue (Festuca arundinacea) decreases cell wall feruloylation and increases rates of cell wall digestion
Source: Plant Cell Tissue Organ Cult. 2017 Feb 7;129(2):181–93. doi: 10.1007/s11240-017-1168-9 (PMC5387028; doi:10.1007/s11240-017-1168-9)
Supplement: Supplementary file 1 — Supplementary material 1 (DOC 5712 KB) [file 11240_2017_1168_MOESM1_ESM.doc]

**Supplementary information**

Expression of a fungal ferulic acid esterase in suspension cultures of tall fescue (*Festuca arundinacea*) decreases cell wall feruloylation and increases rates of cell wall digestion.

Plant Cell Tissue and Organ Culture

Phillip Morris, Sue Dalton, Tim Langdon, Barbara Hauck, and Marcia M. de O. Buanafina

Institute of Grassland and Environmental Research, Plas Gogerddan, Aberystwyth.

Corresponding Author : morrisp14@hotmail.co.uk

**Fig S1** Effect of removal (del-NPIR) or mutation (NPGR) of the NPIR vacuolar targeting motif from the aleurain signal sequence, to convert from vacuole to apoplast targeting, on transient expression of FAEA in *Festuca* cell cultures.

**Fig S2**

Effect of vector orientation of actin-FAE gene and CaMV-hyg gene on transient expression of FAEA in *Festuca* cell suspensions

**Fig S3**

Effects of restoration of a 5bp deletion near the NCO splice site relative to the published sequence and removal of a 400bp repetitive element from the rice actin promoter on transient expression of sgpf in suspension culture cells of *Festuca.* Bombarded plates were examined after 24h.

# Fig S4

Effects of translationally fused forms of FAE with sgfp at the N and C terminus on transient expression of sgfp in suspension culture cells of *Festuca.* Bombarded cells were examined after 48h
